# Characterization of porcine extraembryonic endoderm cells

**DOI:** 10.1111/cpr.12591

**Published:** 2019-03-21

**Authors:** Qiao‐Yan Shen, Shuai Yu, Ying Zhang, Zhe Zhou, Zhen‐Shuo Zhu, Qin Pan, Shan Lv, Hui‐Min Niu, Na Li, Sha Peng, Ming‐zhi Liao, Hua‐Yan Wang, An‐Min Lei, Yi‐Liang Miao, Zhong‐Hua Liu, Jin‐Lian Hua

**Affiliations:** ^1^ College of Veterinary Medicine, Shaanxi Centre of Stem Cells Engineering and Technology Northwest A&F University Yangling China; ^2^ Institute of Stem Cell and Regenerative Biology, College of Animal Science and Veterinary Medicine Huazhong Agricultural University Wuhan China; ^3^ Key Laboratory of Animal Cellular and Genetic Engineering of Heilongjiang Province, College of Life Science North‐East Agricultural University Harbin China

**Keywords:** culture system, extraembryonic endoderm cells, induced pluripotent stem cells, pig, pluripotent

## Abstract

**Objectives:**

To date, many efforts have been made to establish porcine embryonic stem (pES) cells without success. Extraembryonic endoderm (XEN) cells can self‐renew and differentiate into the visceral endoderm and parietal endoderm. XEN cells are derived from the primitive endoderm of the inner cell mass of blastocysts and may be an intermediate state in cell reprogramming.

**Materials and methods:**

Porcine XEN cells (pXENCs) were generated from porcine pluripotent stem cells (pPSCs) and were characterized by RNA sequencing and immunofluorescence analyses. The developmental potential of pXENCs was investigated in chimeric mouse embryos.

**Results:**

Porcine XEN cells derived from porcine pPSCs were successfully expanded in N2B27 medium supplemented with bFGF for least 30 passages. RNA sequencing and immunofluorescence analyses showed that pXENCs expressed the murine and canine XEN markers *Gata6*, *Gata4*, *Sox17* and* Pdgfra* but not the pluripotent markers* Oct4*, *Sox2* and TE marker *Cdx2*. Moreover, these cells contributed to the XEN when injected into four‐cell stage mouse embryos. Supplementation with Chir99021 and SB431542 promoted the pluripotency of the pXENCs.

**Conclusions:**

We successfully derived pXENCs and showed that supplementation with Chir99021 and SB431542 confer them with pluripotency. Our results provide a new resource for investigating the reprogramming mechanism of porcine‐induced pluripotent stem cells.

## 1 INTRODUCTION

Extraembryonic endoderm (XEN) cells are derived from the primitive endoderm of the inner cell mass of blastocysts. XEN cells can self‐renew and differentiate into the visceral endoderm (VE) and parietal endoderm (PE) of the yolk sac.^1^ Murine XEN cells can form flat, stem‐cell‐like colonies with self‐renewal capacity that express* Gata6*, *Pdgfra*, *Sox17*, *Gata4* and *Sox7*.^2^

Extraembryonic endoderm cells have been traditionally derived from blastocysts or post‐implantation embryos.^3^, ^4^ More recent studies have derived XEN cells from embryonic stem (ES) cells or fibroblasts.^5^, ^6^, ^7^, ^8^, ^9^ Murine and canine XEN cell lines maintained in vitro represent a PrE lineage.^1^, ^10^ During cell reprogramming, XEN‐like cells may form during the transition of somatic cells to induced pluripotent stem cells.^11^, ^12^ Two master genes of pluripotency, *Sall4* and *Lin28a*, are expressed during the intermediate XEN‐like cells state. These genes may play an important role in the transition of the XEN‐like state to the pluripotent state.

Pigs are an ideal model for many human diseases and a potential source for organ transplantation. However, pre‐implantation development in pigs is different from that of mice and humans, and the derivation of naïve porcine pluripotent stem cells (pPSCs), from which XEN cells can potentially be derived, is also more challenging. For example, expression patterns of*Oct4*, *Nanog* and *Sox2* in the zona‐enclosed porcine blastocyst are different from those in murine and human blastocysts.^13^ Thus, available lines of naïve pPSCs and porcine XEN cells (pXENCs) remain limited.

In this study, we generated a stable pXENC line as a tool to investigate the characteristics of naïve pPSCs. Our results provide a new resource for investigating cell reprogramming mechanisms and for advancing regenerative medicine research.

## 2 MATERIALS AND METHODS

### 2.1 Culture of porcine pluripotent stem cells (pPSCs)

Porcine pluripotent stem cells were derived from porcine embryos and were maintained on mitomycin‐treated mouse embryonic fibroblasts (feeder cells) and cultured in pPSCs medium.^14^ This consisted of 38% konckout Dulbecco's modified Eagle's medium (DMEM; Gibco, Grand Island, NE, USA), 24% DMEM/F12 (Gibco), 24% Neurobasal (Gibco) and 10% KOSR (Gibco), supplemented with 100 units/mL penicillin‐streptomycin, 0.25% N2 (Gibco), 0.5% B27 (Gibco), 0.25 mg/mL BSA, 1% l‐glutamine, 0.1% β‐mercaptoethanol (Gibco), 40 μg/mL vitamin C (VC; Sigma, St. Louis, MO, USA), 5 ng/mL human Leukemia Inhibitory Factor (LIF) (Sino Biological, Beijing, China) and 8 ng/mL human basic fibroblast growth factor (bFGF) (Sino Biological).^1^ The medium was changed daily. The cells were cultured in humidified conditions with 5% O_2_, 5% CO_2_ and 90% N_2_ at 39°C. Moreover, 1 mg/mL collagenase IV (Gibco) was used to passage cells every 5‐7 days using a split ratio of 1:5.

### 2.2 Culture and treatments of porcine extraembryonic endoderm cells (pXENCs)

Porcine XEN cells were maintained on feeder cells (1 × 10^4^ cells/cm^2^) and cultured in pXENCs medium that consisting of knockout DMEM (Gibco), 100 units/mL penicillin‐streptomycin, 0.25% N2, 0.5% B27, 0.25 mg/mL BSA, 1% l‐glutamine, 0.1% β‐mercaptoethanol and 10 ng/mL human bFGF (Sino Biological). The medium was changed daily. Three to six days the cells were digested into single cells with TrypLE™ (Gibco). The cells were cultured for 30 passages using split ratios from 1:3 to 1:10.

For improved pluripotent of pXENCs, conversion was usually conducted on Day 3 after the passage of pXENCs, and pXENCs colonies usually reached 40%‐50% of confluence. Mitomycin C (Roche, Basel, Switzerland)‐inactivated feeder cells were seeded (3 × 10^4 ^cells/cm^2^) 1 day before the conversion. Small molecules and cytokines were supplemented as indicated at the following: hbFGF (Sino Biological), 10 ng/mL; Chir99021 (Selleck, Houston, Texas, USA), 6 nmol/L; and SB431542 (Selleck), 2 nmol/L. The medium was changed daily. Dome‐shaped colonies gradually emerged during this period. Then, 3‐6 days later, the porcine ES (pES)‐like cells could be digested into single cells with TrypLE™ Select (Gibco). The split ratio was usually from 1:3 to 1:10.

Porcine XEN cells were plated in the pXENCs medium supplemented with 1.0 μmol/l all‐trans retinoic acid (RA) (Sigma) to detect the differentiation ability. Culture medium and RA were changed daily and pXENCs were passaged every 3‐5 days at a 1:3‐1:5 ratio according to cell density.

### 2.3 Culture of porcine‐induced pluripotent stem cells (piPSCs)

Porcine‐induced pluripotent stem cells (piPSCs) generated by inducing forcing the expression of doxycycline‐inducible OSKM factors in fibroblasts.^15^ The piPSCs cultured in DMEM supplemented with 15% foetal bovine serum (FBS; Gibco), 10 ng/mL human LIF (Sino Biological), 10 ng/mL bFGF (Sino Biological), 3 nmol/L/mL Chir99021 (Selleck) and SB431542 (Selleck), 4 µg/mL Dox (Sigma). The TrypLE™ Select (Gibco) was used to passage piPSCs every 3 days. The split ratio was usually from 1:20 to 1:30.

### 2.4 Gene expression analyses

RNA was isolated by Trizol (Invitrogen, Carlsbad, CA, USA) extraction in accordance with the manufacturer's instructions.^16^ The quality of RNA samples was determined on the basis of 260/280 ratio. cDNA was synthesized by using the FastKing RT Kit (Tiangen, Beijing, China). Quantitative real‐time PCR (qPCR) analyses were performed using the SuperReal Color PreMix (Tiangen) in biological triplicate. All qPCR primers used are listed in Table S1. Each qRT‐PCR reaction included 10 μL SYBR® Premix Ex Taq II (2×), 0.8 μL cDNA, 0.5 μL PCR Forward Primer (10 μmol/L), 0.5 μL PCR Reverse Primer (10 μmol/L) and sterile water to a total volume of 20 μL, and involved denaturation at 95°C for 3 minutes followed by 40 cycles of (95°C for 15 seconds, 60°C for 30 seconds, 72°C for 30 seconds).

### 2.5 RNA sequencing and analysis

Total RNA was extracted from piPSCs and pXENCs using Trizol (Invitrogen) reagent. For RNA‐seq, sequencing libraries were created from each group using the NEBNext® Ultra™ Directional RNA Library preparation kit (Illumina, San Diego, CA, USA). Briefly, total RNA was fragmented into small pieces using divalent cations at elevated temperature. The cleaved RNA fragments were copied into first‐strand cDNA using reverse transcriptase and random primers, followed by second‐strand cDNA synthesis using DNA polymerase I and RNase H. After adenylation of 3′ ends of DNA fragments, NEBNext Adaptor with hairpin loop structure was ligated to prepare for hybridization. In order to select cDNA fragments of preferentially 150‐200 bp in length, the library fragments were purified with AMPure XP system (Beckman Coulter, CA, USA). Then, PCR was performed with Phusion High‐Fidelity DNA polymerase, Universal PCR primers and Index (X) Primer. At last, PCR products were purified (AMPure XP beads). The cDNA fragments were sequenced using the Illumina HiSeq at Mega Genomics. The RNA‐seq reads were aligned to pig genome (Sscrofa11.1) using Tophat2 alignment software with default.^17^ Gene expression level was measured as fragments per kilobase million.^18^

Differentially expressed genes were detected by the package DEseq in the R software.^19^ An adjusted *P* value <0.05 and an absolute value of the log_2_ ratio >1 were used as the threshold for declaring gene expression differences as being significant. Heatmaps were generated using heatmap package in the R software. For the gene ontology analysis of the differentially expressed genes, gene lists were subjected to DAVID bioinformatics tool.^20^ Terms that had a *P* value of <0.05 were defined as being significantly enriched. The genes are classified according to expression pattern of marker genes and developmental cell identity using LifeMap Diccovery.^21^

### 2.6 Alkaline phosphatase staining

Alkaline phosphatase (AP) activity was detected by using AST Fast Red TR (Sigma) and α‐Naphthol AS‐MX Phosphate (Sigma) in accordance with the manufacturer's protocol. Cells were washed with phosphate‐buffered saline (PBS) and fixed with 4% paraformaldehyde in PBS for 15 minutes at room temperature. The fixed cells were washed once with PBS and incubated with the mixture at room temperature for 15 minutes.^15^ The cells were observed and the images were captured by using a Nikon (Tokyo, Japan) inverted microscope after staining.

### 2.7 Interspecies chimera generation

Mouse embryos at the two‐cell stage were recovered from 1‐day postcoitus oviducts and cultured to the four‐cell stage in KSOM medium. The cultured pXENCs were labelled with fluorescent dye (PKH26 Red Fluorescent Membrane Linker; Sigma). Approximately, eight pXENCs were microinjected into four‐cell stage mouse embryos to produce interspecies chimeric embryos. The embryos were cultured to blastocyst stage in KSOM medium. Each individual blastocyst was then placed onto MEFs in a well of a four‐well dish coated with 0.1% gelatin. Blastocysts began to form outgrowths after 3 days of culture. The embryos were subjected to immunofluorescence staining after 10 days.

### 2.8 Embryoid body formation assay

Porcine XEN cells and piPSCs were digested into single cells, which were separated from MEF feeder cells through preplating on gelatin‐coated plates, and cultured for 7 days on ultralow attachment plates in IMDM supplemented with 15% FBS. Embryoid bodies (EBs) were collected and plated for 7 days in the same medium, fixed, and subjected to analysis.

### 2.9 Immunofluorescence

The cells were fixed in 4% paraformaldehyde (AR‐0211; DingGuo, Beijing, China) for 15 minutes at room temperature and blocked with PBS that contained 0.2% Triton X‐100 (T8787; Sigma‐Aldrich) and 5% normal donkey serum for 45 minutes at room temperature. The cells were incubated with primary antibodies at 4°C overnight. Afterwards, secondary antibodies were incubated for 1 hour at room temperature. The nucleus was stained with Hoechst33342 (Sigma). Primary antibodies were used against GATA4 (#BM5082; Boster Biological Technology, Wuhan, China) or against GATA6 (#AF1700), SOX17 (#AF1924) or PDGFRa (#AF1062; all from R&D Systems, Minneapolis, MN, USA). Secondary antibodies were Alexa Fluor 488 AffiniPure Donkey Anti‐Goat IgG (H + L; #705‐545‐147; Jackson ImmunoResearch Laboratories, West Grove, PA, USA) and Alexa Fluor® 488 conjugate goat anti‐rabbit IgG (H + L; #ZF‐0511; ZSGB‐BIO, Beijing, China).^22^

### 2.10 Statistical analysis

Each experiment included independent triplicate samples. All data are shown as mean ±  SEM. Student's *t* test was used to identify significant mean differences between two groups. One‐way or two‐way ANOVA were used to compare means among three or more independent groups. A value of *P* < 0.05 was considered significant. Statistical analyses were performed using Prism Software (Prism Software, San Diego, CA, USA).

## 3 RESULTS

### 3.1 pXENCs

**Figure 1:**
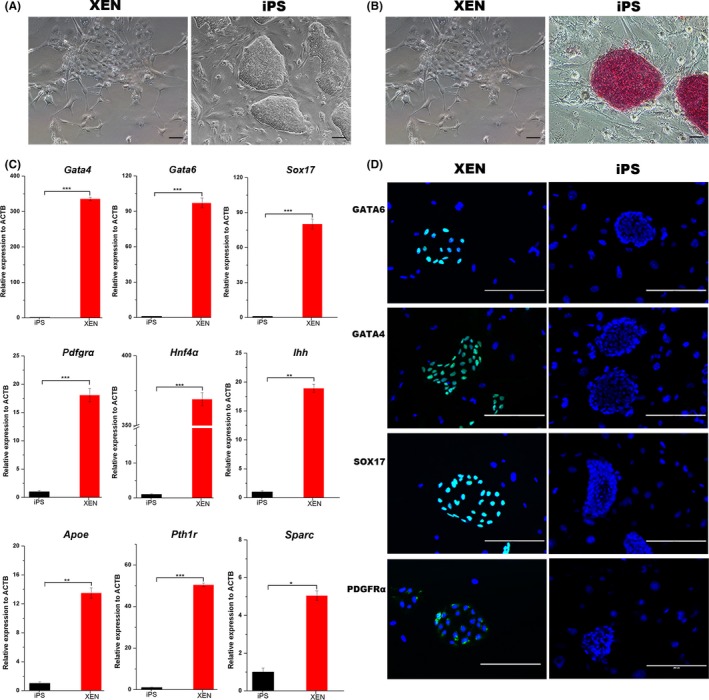
Derivation and characterization of porcine extraembryonic endoderm cells (pXENCs). A, Formation of pXENCs (left) and of porcine‐induced pluripotent stem cells (piPSCs; right) during cultivation. B, Alkaline phosphatase (AP)‐negative pXENCs (left) and AP‐positive piPSCs (right). C, Quantitative real‐time PCR analysis for XEN cells markers (*Gata4*, *Gata6*, *Sox17*, *Pdfgra*, *Hnf4a*, *Ihh*, *Apoe*, *Pth1r* and *Sparc*) in pXENCs and piPSCs. Data are represented as mean ± SD. *P < 0.05,**P<0.01 and ***P < 0.001. D, Immunofluorescence images showing the presence/absence (in green) of GATA6, GATA4, PDGFRα and SOX17 in pXENCs (left) and piPSCs (right). Scale bars: 100 µm (A), 50 µm (B), 200 µm (D)

Distinct colonies with irregular borders appeared when pPSCs were cultured in pXENCs medium. Colonies were picked and digested into single‐cell suspensions before passage. Clones derived from single cells could be expanded for at least 30 passages (Figure 1A). qPCR and immunofluorescence analyses confirmed these clones to correspond to pXENCs. Compared with piPSCs, the pXENCs were AP negative (Figure 1B) and did not express pluripotent markers including *Oct4*, *Sox2*, c‐*Myc* and *Klf4*. pXENCs expressed XEN markers, such as *Gata4*, *Gata6*, *Sox17*, *Pdgfra*, *Hnf4a*, *Ihh*, *Apoe*, *Pth1r* and *Sparc*, at significantly higher levels than piPSCs (Figure 1C). Immunofluorescence showed the presence in pXENCs of GATA6, GATA4, SOX17 and PDGFRa but not OCT4 and SOX2 (Figure 1D).

To identify the factors responsible for the maintenance of pXENCs in culture, we sequentially removed bFGF, LIF and VC from the culture medium. We found that cell proliferation was arrested in the absence of bFGF, but was unaffected in the absence of LIF and VC (Figure S1A,B). The expression of XEN markers markedly increased in the absence of LIF. Cultured pXENCs exhibited two different morphologies, dispersed and aggregated (Figure 1A). However, further analyses found no differences in gene expression levels associated with the two morphologies (Figure S1C). pXENCs became senescence, forming large, flat clones when N2B27 was replaced with 5% KSR and 5% FBS (Figure S1D).

### 3.2 RNA‐seq profiles of pXENCs

**Figure 2:**
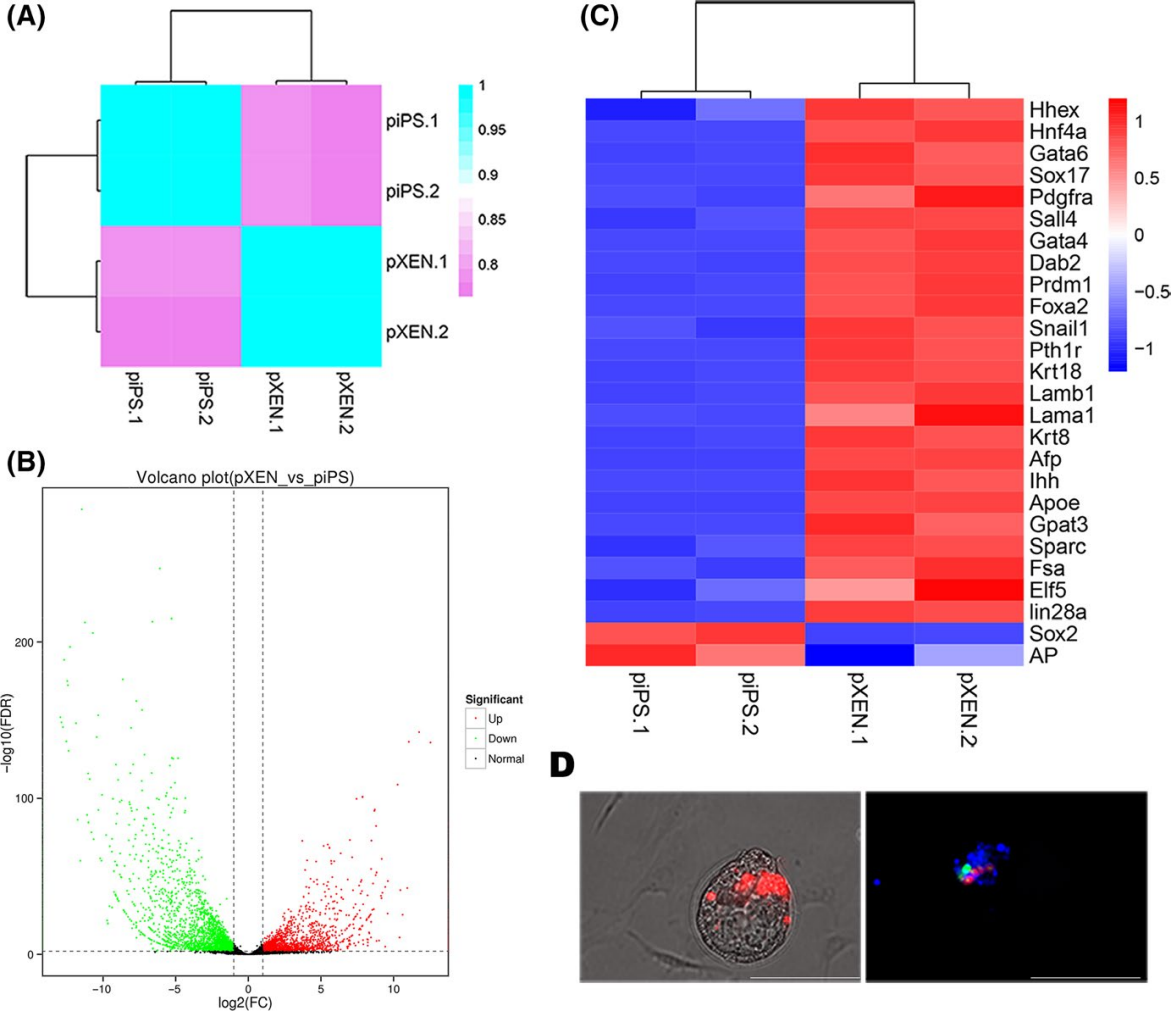
RNA sequencing and chimera analyses. A, Pearson's correlation coefficients obtained from RNA sequencing of porcine extraembryonic endoderm cells (pXENCs) and porcine‐induced pluripotent stem cells (piPSCs). B, Volcano and C, Heatmap cluster plots showing differences in gene expression between pXENCs and piPSCs. D, Whole mount of a chimeric embryo imaged under bright field (left) and fluorescence (right) by using a Nikon inverted microscope. Hoechst33342 (blue), GATA4 (green) and pXENCs (red). Scale bars: 100 µm (D)

Pearson's correlation coefficients obtained from RNA sequencing data indicated highly reproducible gene expression patterns between samples for each of piPSCs and pXENCs (Figure 2A). Compared with piPSCs, pXENCs had 2793 upregulated genes and 1909 downregulated genes among the 17 675 identified genes (Figure 2B, Figure S2; Table S2). pXENCs expressed typical XEN markers, while lacking pluripotency‐related genes, such as *Sox2* and AP (Figure 2C, Table S3). We further examined the ability of pXENCs to contribute to early development after injection into four‐cell stage mouse embryos. Figure 2D shows GATA4‐expressing chimeric cells confirming they are pXENCs.

### 3.3 Differentiation potential of pXENCs

**Figure 3:**
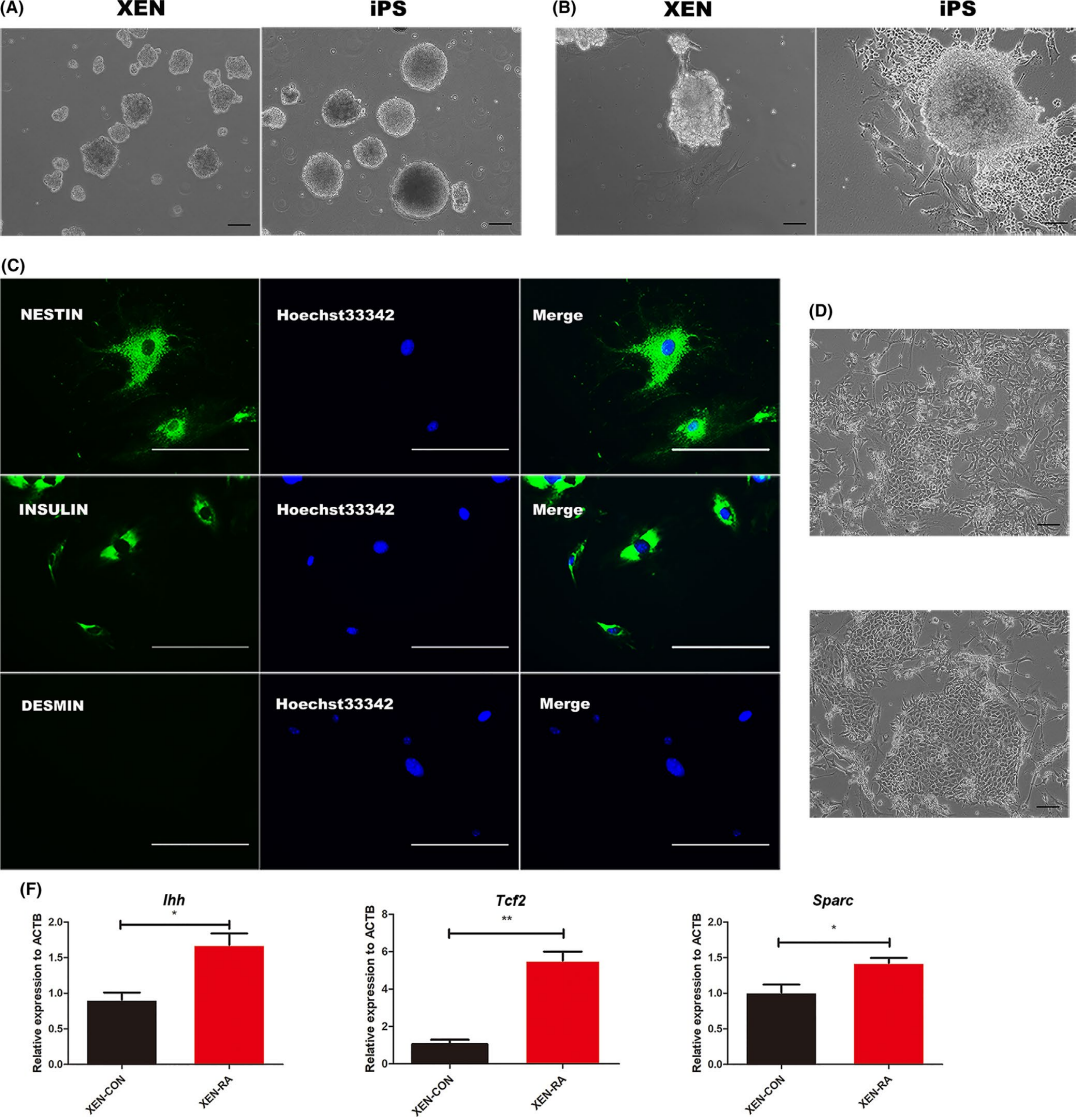
Differentiation capacity of porcine extraembryonic endoderm cells (pXENCs). A, Phase images of embryoid bodies (EBs) suspensions of pXENCs and porcine‐induced pluripotent stem cells (piPSCs) after 4 d in EBs conditions. B, Fewer pXENCs than piPSCs migrated from attached EBs. C, Immunofluorescence of pXENCs‐derived EBs for markers of endoderm (INSULIN), ectoderm (NESTIN) and mesoderm (DESMIN). D, When the pXENCs were passaged by single cells, the cells were dispersed after 2 d. E, Cells tended to aggregate in the presence of RA. F, Quantitative real‐time PCR analysis of XEN markers in pXENCs treated with RA. Data are represented as mean ± SD. *P < 0.05 and **P < 0.01. Scale bars: 100 µm (A, D, E), 50 µm (B), 200 µm (C)

Extraembryonic endoderm cells were able to form EBs in suspension culture. EBs that formed from pXENCs exhibited a more irregular morphology than those derived from piPSCs (Figure 3A,B). pXENCs derived EBs differentiated into cells that expressed markers of ectoderm and endoderm (NESTIN and INSULIN) but not mesoderm (DESMIN).

RA induces differentiation of XEN cells.^23^ In culture, pXENCs exhibited dispersed morphology (Figure 3D) but became highly aggregated after the addition of RA (Figure 3E). After RA addition, the levels of XEN markers, such as *Gata4*, *Gata6,* *Sox17* and *Pdgfra*, remained unchanged compared with those in the control group that without RA addition. However, the expression of the PE and VE markers, such as* Ihh*,* Tcf2* and *Sparc*, increased in pXENCs relative to the group of without RA addition after RA addition.

### 3.4 Promotion of pXENCs pluripotency

**Figure 4:**
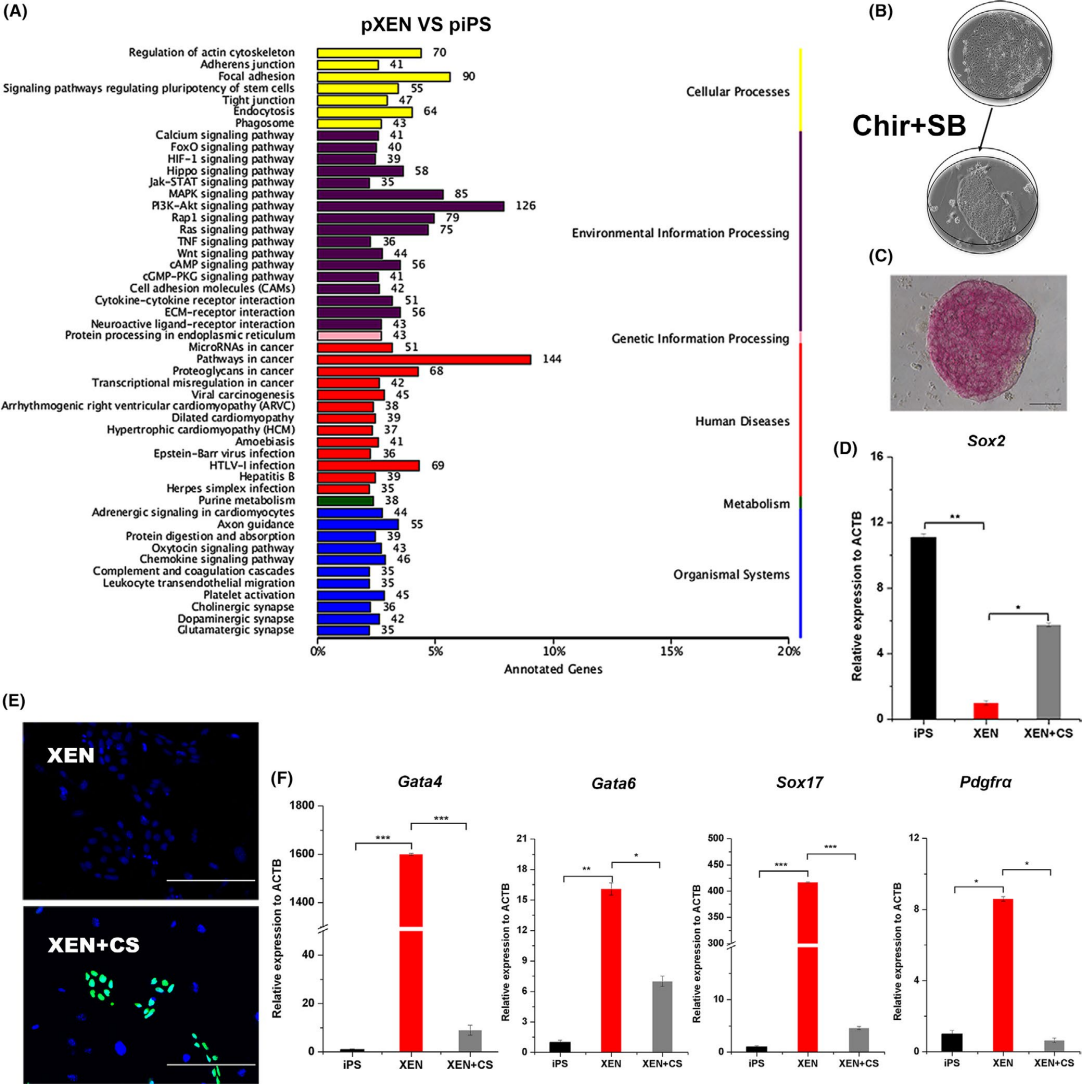
Promotion of porcine extraembryonic endoderm cells (pXENCs) pluripotency. A, Signalling pathway enrichment in gene subsets differentially expressed between pXENCs and porcine‐induced pluripotent stem cells (piPSCs). B, Phase images and C, Alkaline phosphatase staining of pXENCs cultures treated with Chir99021 and SB431542. D, Quantitative real‐time PCR (qRT‐PCR) analysis of *Sox2 *in piPSCs and pXENCs treated with Chir99021and SB431542 (XEN + CS). Data are represented as mean ± SD. *P < 0.05 and **P < 0.01. E, Immunofluorescence images showing staining of SOX2 in pXENCs untreated or treated with Chir99021 and SB431542 (XEN + CS). F, qRT‐PCR analysis of XEN markers in piPSCs, pXENCs and pXENCs treated with Chir99021 and SB431542 (XEN + CS). Data are presented as mean ± SD. *P < 0.05 **P<0.01 and ***P < 0.001. Scale bars: 100 µm (C), 200 µm (E)

Genes differentially expressed between pXENCs and piPSCs were enriched for a number of different cell signalling pathways, as shown in Figure 4A. We tested whether the two small‐molecule compounds Chir99021 and SB431542 were able to reverse pXENCs to a pluripotent state (Figure 4B). AP activity and endogenous *Sox2* expression increased in pXENCs in the presence of the two inhibitors (Figure 4C‐E). Moreover, the expression of XEN cell markers (*Gata4, Gata6, Sox17 and Pdgfra*) significantly decreased in response to treatment with Chir99021 and SB431542 (Figure 4F). These results showed that Chir99021 and SB431542 promote pluripotency of pXENCs.

## 4 DISCUSSION

Cultures of ES and XEN cells can be obtained from epiblast and primitive endoderm, respectively. Gene expression patterns, differentiation potential and lineage restriction are maintained in XEN cells, therefore providing a useful model to study primitive endoderm.^24^ ES cells can differentiate into somatic and extraembryonic lineages in vitro including trophectoderm and XEN cells^25^; however, a stable culture system for pES cells is currently unavailable. The culture system we used in this study is very different from that used to culture murine XEN cells,^10^ but was highly effective for deriving and expanding pXENCs, allowing these cells to maintain XEN characteristics for at least 30 passages. pXENCs colonies were flatter and looser (allowing observation of individual cells) than piPSCs colonies.

We found 4702 differentially expressed genes between pXENCs and piPSCs. Many typical XEN markers, such as *Gata6*,* Gata4*, *Sox17* and *Pdgfra, *were upregulated in XEN cells, whereas *Sox7 *was almost undetectable. Future work we should investigate the function of *Sox7* in pXENCs.^3^

Extraembryonic endoderm‐like cells were an intermediate state during cell reprogramming.^12^ Mouse XEN cells express *Lin28a*, *Sall4* and other pluripotent genes. iPS cells can be rapidly obtained by changing culture conditions or by overexpressing *Oct4 *and* Sox2* in XEN cells.^12^ bFGF signalling is important to maintain human and bovine ES cells in culture.^26^, ^27^ pXEN cell obtained in our study depended on bFGF for growth and expressed *Lin28a*, *Sall4* and other pluripotency genes. Therefore, pXENCs may have the same potential as murine XEN cells and may easily transition into iPS cells. Thus, following on the identification of differences in signalling pathways between pXENCs and piPSCs, we screened for small molecules that could promote pluripotency in pXENCs. We found that treatment of pXENCs with Chir99021 and SB431542 for 4 days resulted in AP‐positive cells expressing high levels of *Sox2*, indicating an ability of pXENCs to transition into PSCs. Given that the establishment of stable pES cell lines has not been reported, developing robust approaches for generation of pES cells from pXENCs may prove extremely useful. Moreover, based on their gene expression patterns, our XEN‐derived pES‐like cells had not reached a naive pluripotent state, because the pluripotent marker, *Oct4* was not expressed, and no mesoderm cells formation during EBs test. Further screening of small‐molecule inhibitors and effector transcription factors may lead to the derivation of bona fide pPSCs.^6^ pXENCs could be also used as an in vitro tool to explore and identify the mechanisms and pivotal molecules involved in cell reprogramming.^10^ A clear advantage of XEN cells over other cell types is that they can be propagated in large quantities in culture media that do not require complex components.^28^, ^29^

To summarize, we have established one pXENC line exhibiting typical morphology and markers as murine XEN cells. pXENCs can be stably maintained in culture and potentially provide a source for the derivation of pES cells in future.
