# Correction to “Characterization of porcine extraembryonic endoderm cells”

**DOI:** 10.1111/cpr.13537

**Published:** 2023-09-04

Shen Q‐Y, Yu S, Zhang Y, et al. Characterization of porcine extraembryonic endoderm cells. *Cell Prolif*. 2019;52:e12591. https://doi.org/10.1111/cpr.12591

Figures 1A and 1B are duplicates. The corrected image for Figure 1 is below:

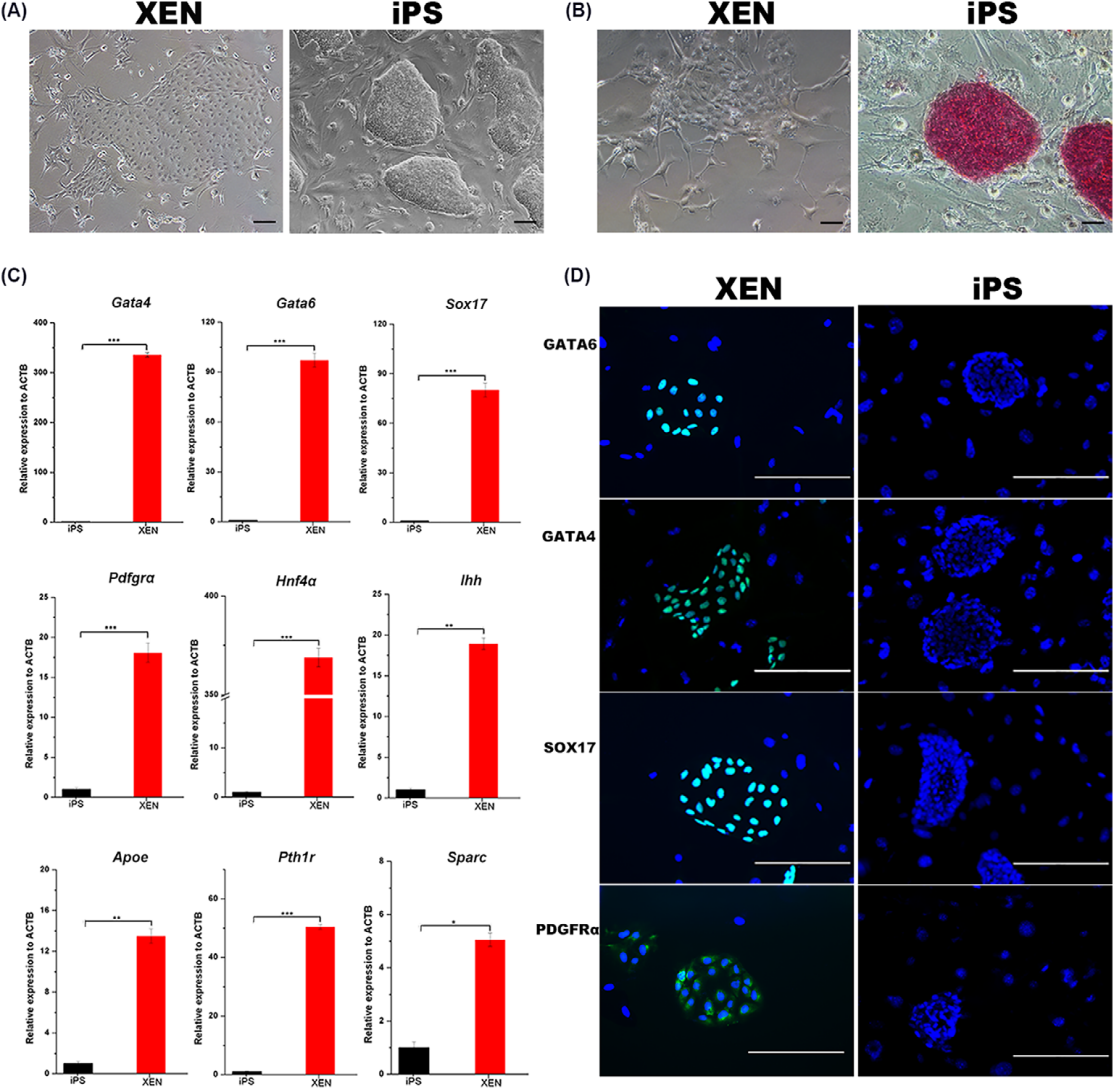

The error does not affect the overall results and conclusions of the study but may lead to confusion regarding the specific cell images.

The online version of this article was corrected.

We apologize for this error.

