# Supplementary material for: Characterization of porcine extraembryonic endoderm cells
Source: Cell Prolif. 2019 Mar 21;52(3):e12591. doi: 10.1111/cpr.12591 (PMC6536407; doi:10.1111/cpr.12591)
Supplement: Supplementary file 1 [file CPR-52-e12591-s001.docx]

**Supplemental information**

**Characterization of porcine extra-embryonic endoderm (XEN) cells**

QiaoYan Shen, Shuai Yu, Ying Zhang, Zhe Zhou, ZhenShuo Zhu, Qin Pan, Shan Lv , HuiMin Niu, Na Li, Sha Peng, MingZhi Liao, HuaYan Wang, AnMin Lei, YiLiang Miao, ZhongHua Liu, JinLian Hua

**SUPPLEMENTAL FIGURES**

**Figure. S1**


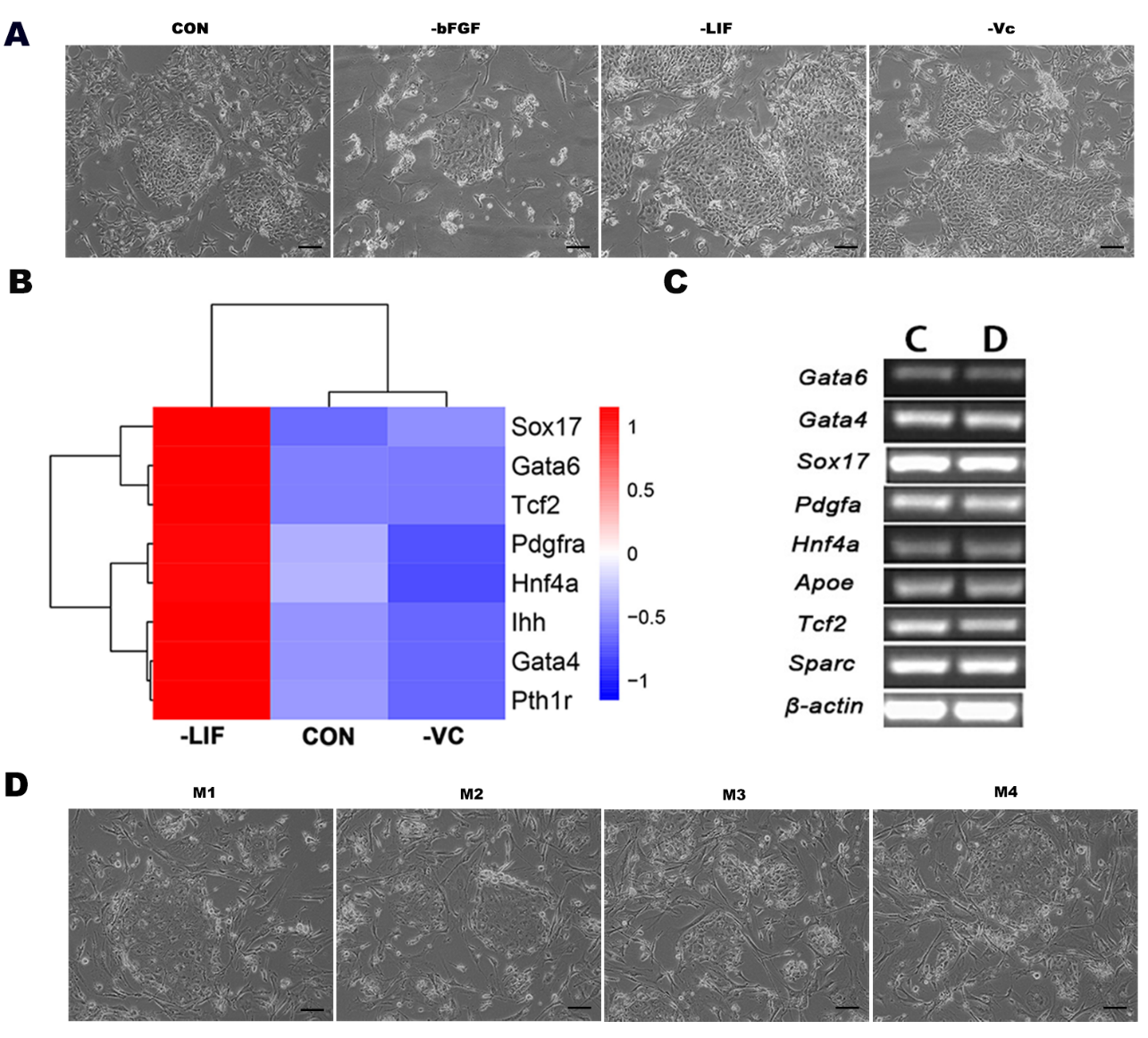


**Figure. S1**

A. The morphology of pXENCs after withdrawing bFGF, LIF and Vitamin C. Scale bar, 100 µm.

B. The detection of expression level of typical pXENCs state markers (withdrawing LIF and Vitamin C)

C. RT-PCR detection the pXENCs markers from loose and tight clones. (C: tight pXENCs clone, D: loose pXENCs clone).

D. pXENCs morphology when used different media of mouse XEN cells. M: Medium; M1: RPMI1640+10% FBS; M2: RPMI1640+10% FBS+25 ng/ml FGF4+1 μg/ml heparin; M3: RPMI1640+10% KSR; M4: PRMI1640+10% KSR+ 25 ng/ml FGF4+1 μg/ml Heparin. Scale bar, 100 µm.

**Figure. S2**


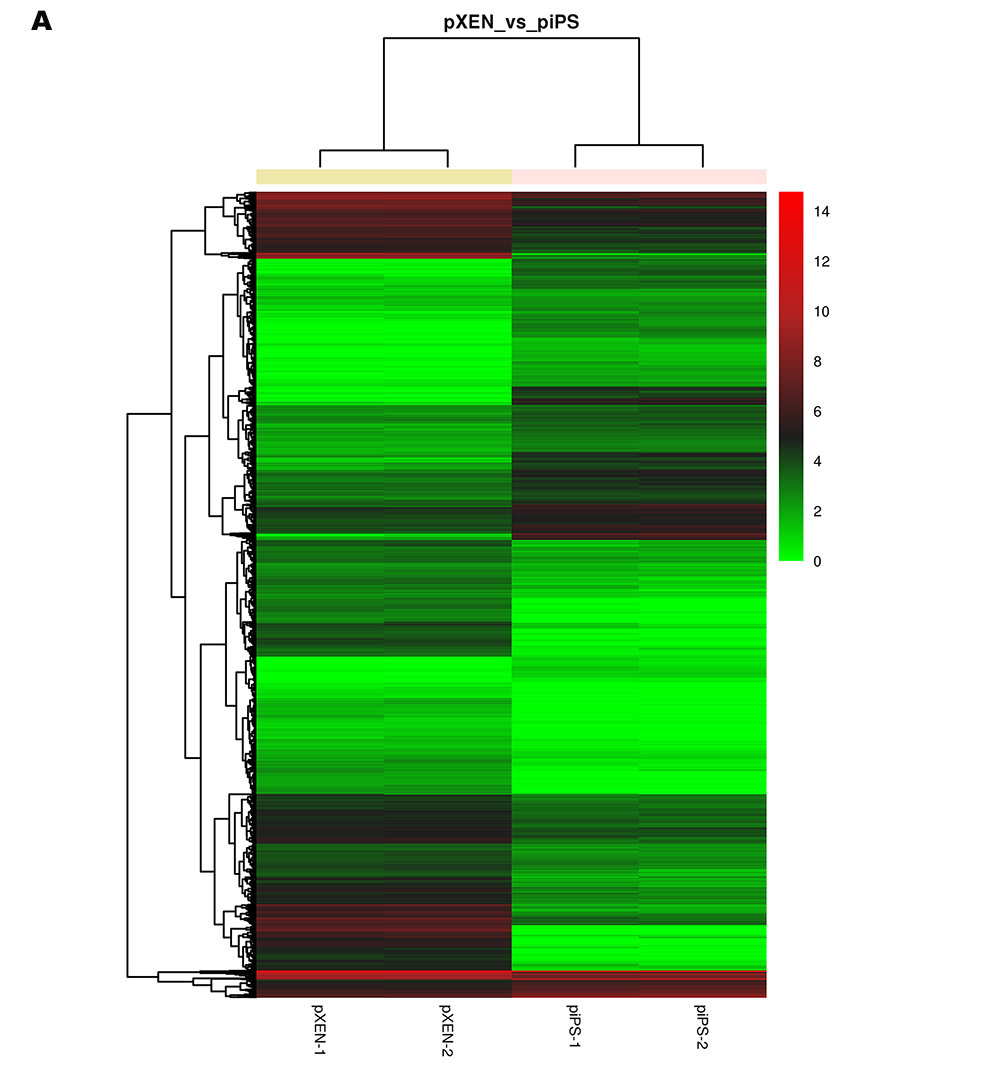


**Figure. S2** A. Heatmap cluster plot of all genes expressed in pXENCs and piPSCs.
