# Supplementary material for: Characterization of porcine extraembryonic endoderm cells
Source: Cell Prolif. 2019 Mar 21;52(3):e12591. doi: 10.1111/cpr.12591 (PMC6536407; doi:10.1111/cpr.12591)
Supplement: Supplementary file 2 [file CPR-52-e12591-s002.docx]

**Table S1.** Primers used for quantitative real-time PCR (qRT-PCR)

| **Target** | **Primer sequences (5′–3′)** | **Length of production/bp** |
| --- | --- | --- |
| *Gata6* | F:GAGCTGGGAGGACCTATTGC | 137 |
|  | R:CTGCGAGGGTCTGGTACATC |  |
| *Gata4* | F:TCCTGTGCCAACTGCCAGACCA | 196 |
|  | R:AGGGACCTGCTGACGTCTTCGAT |  |
| *Sox17* | F:GACCGCACGGAGTTTGAACAA | 150 |
|  | R:GTAATACACTGCGGAGCTGGCAT |  |
| *Pdgfra* | F:ATCCTGGACAAGTGAAAGGCAAAG | 134 |
|  | R:GCGGGCAGCACATTCGTAA |  |
| *Hnf4a* | F:CGCAGGTCAAGCTACGAGGATA | 141 |
|  | R:ATCTGCGATGCTGGCGATCT |  |
| *Hhex* | F:GACTGTCCATCCCATCCATTCT | 152 |
|  | R:GAAGGCAGACAGCAAACCCT |  |
| *Pth1r* | F:CTGAGTCTGGGGAGGACACT | 159 |
|  | R:GGTAGGCGTGGCCTTTATGA |  |
| *Sparc* | F:GCTCTCGCCTAAACCCAGTT | 179 |
|  | R:ACCCCTGTCGGATGTAGTGA |  |
| *Apoe* | F:CTGTGGGTTGCTTTGGTGGT | 154 |
|  | R:GCAGGTAATCCCAGAAGCGG |  |
| *Ihh* | F:GATCGCGACCGGAACAAGTA | 111 |
|  | R:CTCGGACTTGACGGAGCAAT |  |
| *Tcf2* | F:GTGCAATTTGGGCCCTCCA | 231 |
|  | R:GCTGGCAGTCAGTCCAAAAA |  |
| *Lin28a* | F: GAAGTCTGCTAAGGGCTTGGAATC | 119 |
|  | R: TGTCTCCCTTGGATCTGCGTTT |  |
| *Sox2* | F:GCAACCAGAAGAACAGCCCAGA | 110 |
|  | R:GTTGTGCATCTTGGGGTTCTCTTG |  |
